# Development of peptidomimetic ligands of Pro-Leu-Gly-NH_2_ as allosteric modulators of the dopamine D_2_ receptor

**DOI:** 10.3762/bjoc.9.24

**Published:** 2013-01-30

**Authors:** Swapna Bhagwanth, Ram K Mishra, Rodney L Johnson

**Affiliations:** 1 Department of Medicinal Chemistry, University of Minnesota, 308 Harvard Street SE, Minneapolis, MN 55455, USA; 2 Department of Psychiatry and Behavioral Neurosciences, McMaster University, 1200 Main Street W, Hamilton, Ontario L8N 3Z5, Canada

**Keywords:** allosteric modulation, dopamine D_2_ receptor, peptidomimetic, Pro-Leu-Gly-NH_2_, spiro-bicyclic scaffold

## Abstract

A variety of stable, small-molecule peptidomimetic ligands have been developed to elucidate the mechanism by which the neuropeptide Pro-Leu-Gly-NH_2_ (PLG) modulates dopaminergic neurotransmission. Photoaffinity labeling ligands based upon PLG peptidomimetics have been used to establish that PLG binds to the D_2_ dopamine receptor at a site that is different from the orthosteric site, thus making PLG and its peptidomimetics allosteric modulators of the dopamine receptor. Through the design, synthesis and pharmacological evaluation of conformationally constrained peptidomimetics containing lactam, bicyclic, and spiro-bicyclic scaffolds, support was provided for the hypothesis that the bioactive conformation of PLG is a type II β-turn. In addition, studies with peptidomimetics designed to mimic either a type VI β-turn or polyproline II helix conformation yielded molecules that were able to modulate dopamine receptors because of their ability to place the carboxamide NH_2_ pharmacophore in the same topological space as that seen in the type II β-turn. Extensive studies with the spiro-bicyclic PLG peptidomimetics also established that both positive and negative modes of modulation were possible for the same series of peptidomimetics simply as a result of minor differences in the stereochemistry about the bridgehead carbon within the scaffold. This information was used to transform existing positive modulators into negative modulators, which demonstrated that small structural changes in the spiro-bicyclic dopamine receptor modulators are capable of causing major changes in the modulatory activity of PLG peptidomimetics.

## Review

There has been an increasing effort to identify molecules that are able to act as allosteric regulators of specific G protein-coupled receptors (GPCRs), since such ligands have the potential to serve as novel therapeutic agents that are able to provide a means of fine-tuning receptor responses to orthosteric agonists or antagonists. In recent years, the identification of allosteric modulators for GCPRs has increased significantly. The adenosine, muscarinic, chemokine, dopamine, serotonin, calcium-sensing, and metabotropic glutamate receptors are just some examples of GPCRs for which allosteric modulators have been reported [1–2].

The neuropeptide Pro-Leu-Gly-NH_2_ (PLG) has been shown to be a positive allosteric modulator of the dopamine D_2_ receptor [3]. PLG was initially isolated from brain tissue in the search for hypothalamic releasing factors, wherein it was found to inhibit the release of melanocyte stimulating hormone from the pituitary gland [4]. Early on, however, it was found that PLG also possessed significant neuropharmacological activity as a modulator of dopaminergic neurotransmission within the CNS [5], as illustrated by its ability to potentiate the behavioral effects of L-DOPA [6], to enhance the affinity of dopamine receptor agonists to dopamine receptors [7], and to prevent neuroleptic drug-induced supersensitivity of post-synaptic dopamine receptors [8]. The molecular basis behind this enhancement of dopaminergic neurotransmission did not become known until several decades later when photoaffinity-labeling peptidomimetics of PLG were used to show that PLG and its peptidomimetics act as allosteric modulators of the dopamine D_2_ receptor [3,9]. This represents one of the few examples where a known endogenous molecule has demonstrated allosteric receptor-modulating activity, since most allosteric modulators discovered to date are exogenous synthetic molecules that have been identified through screening protocols and then subsequently optimized through structural modification [2].

Although PLG’s pharmacological profile suggested that this compound would have potential in treating neurological diseases such as Parkinson’s disease and tardive dyskinesia, the peptide nature of PLG limits its potential as a drug [10–11]. Thus, efforts were initiated to design peptidomimetic analogues of PLG in the hope of developing agents that would possess the same activity as PLG, but have better pharmacokinetic properties. As described below, these efforts have led to peptidomimetics that have helped elucidate the bioactive conformation of PLG, as well as the mechanism by which these compounds positively modulate dopamine receptors. In addition, these efforts have led to the discovery of peptidomimetics that negatively modulate dopamine receptors.

### PLG peptidomimetic design

**Figure 1:**
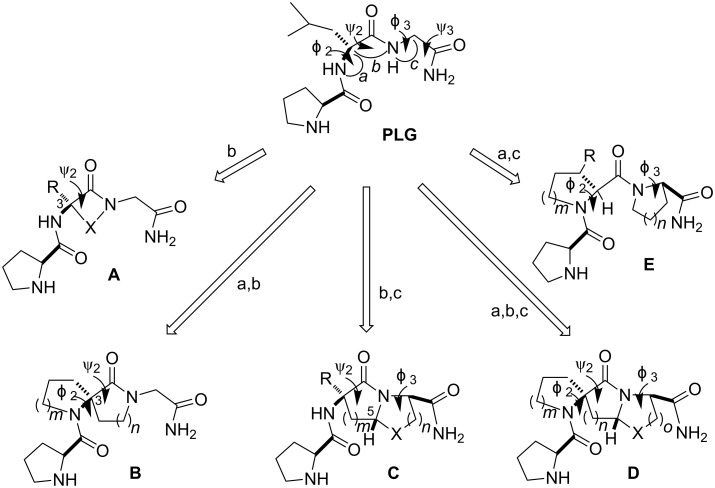
PLG peptidomimetic design approach. The Φ_2_, ψ_2_, Φ_3_, and ψ_3_ torsion angles define the postulated β-turn structure of PLG and a–c represent bridging connections made between the designated atoms of the peptide backbone to generate the peptidomimetic analogues **A**–**E** of PLG.

Although several studies of structure–activity relationships on PLG had been carried out early on [12–14], these studies did not provide information about the conformation PLG adopts to produce its pharmacological actions. NMR spectroscopic studies [15] and computational studies [16] indicated that PLG, although a flexible molecule, assumes turn conformations. The X-ray crystal structure of PLG showed that it existed in a type II β-turn [17]. We began our PLG peptidomimetic development studies by trying to understand what is the bioactive conformation of PLG. Our working hypothesis was that PLG exists in a type II β-turn conformation when it produces its effects. To test this hypothesis, a series of conformationally constrained analogues of PLG were designed that would restrict PLG in such a conformation. The overall approach employed is summarized in Figure 1 and it involved the incorporation of one or more bridging units between the atoms of the peptide backbone, thereby constricting one or more of the four torsion angles (Φ_2_, ψ_2_, Φ_3_, and ψ_3_) that define the postulated turn structure of PLG.

### Lactam PLG peptidomimetics

**Figure 2:**
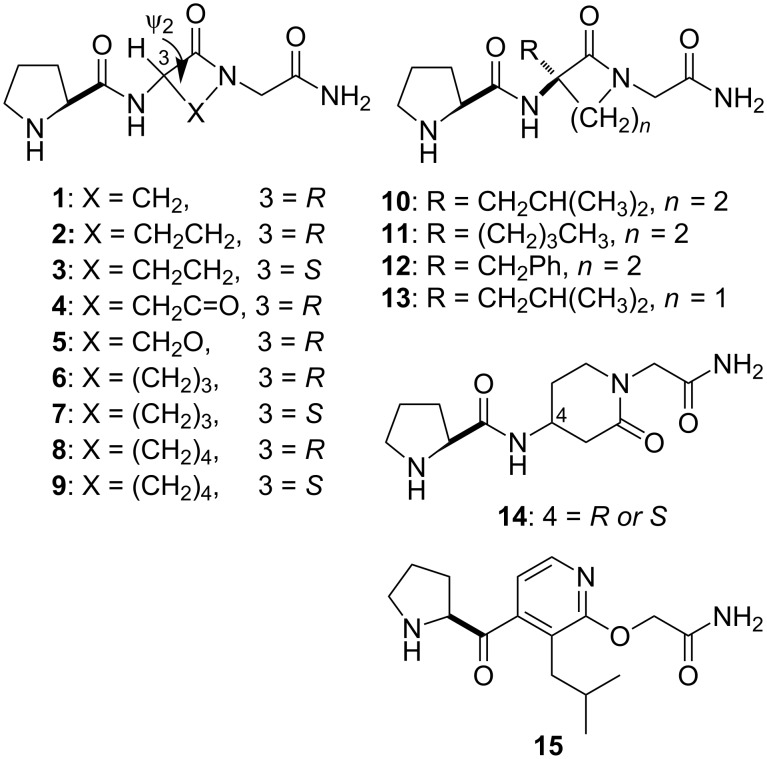
Lactam-based PLG peptidomimetics.

The first confomationally constrained analogues of PLG synthesized incorporated the lactam approach developed by Freidinger et al. [18] to provide PLG peptidomimetics of type **A** (Figure 1). The lactam scaffold constrains the ψ_2_ torsion angle, the value of which varies depending upon the nature of X. In addition, the type of β-turn that is mimicked is dictated by the chirality of C-3. Lactams **1**, **2**, **4**–**6**, and **9** (Figure 2) were active in enhancing the binding of the dopamine receptor agonist 2-amino-6,7-dihydroxy-1,2,3,4-tetrahydronaphthalene (ADTN) to dopamine receptors, while **3**, **7**, and **8** were inactive [19–20]. The activity seen with **2** and the inactivity of **3** supported the hypothesis that the bioactive conformation of PLG was a type II β-turn, since the (*R*)-isomer of the γ-lactam mimics a type II β-turn, while the (*S*)-isomer supports a type II’ β-turn structure. Although an X-ray structure of **2** showed that the γ-lactam constraint restricted the ψ_2_ torsion angle to 141.9°, i.e., a value close to the 120° seen in an ideal type II β-turn [21], **2** did not exist in a type II β-turn conformation in the crystal state. The same dependence on chirality was seen with δ-lactam analogues **6** and **7**. In contrast, in the case of the ε-lactam analogues **8** and **9**, it is the lactam with the (*S*)-chirality that possesses the activity. This was expected, as previous studies had shown that the (*R*)-ε-lactam restricts the ψ_2_ torsion angle to around −168°, while the (*S*)-ε-lactam restricts the ψ_2_ torsion angle to around +168°. This positive ψ_2_ value is consistent with the positive ψ_2_ value required of an ideal type II β-turn [22].

In the series of lactam PLG peptidomimetics shown in Figure 2, peptidomimetic **2** was found to be the most potent in enhancing the binding of the dopamine receptor agonist ADTN to isolated dopamine receptors. It was over a 1000-fold more potent than PLG [19]. Like PLG, this enhanced binding of agonists to the dopamine receptor produced by **2** was brought about by increasing the affinity of agonists to the receptor and by shifting the ratio of low- and high-affinity states of the dopamine receptor to the high-affinity state, which couples to the G-proteins [23]. Studies carried out in cell lines transfected with human dopamine receptor subtypes have shown that PLG and **2** enhance agonist binding to the D_2S_, D_2L_ and D_4_ dopamine receptor subtypes, whereas the D_1_ and D_3_ receptor subtypes are unaffected [24]. Peptidomimetic **2** was also more potent than PLG in in vivo assay systems, including (1) potentiation of apomorphine-dependent rotational behavior in 6-hydroxydopamine lesioned rats [25]; (2) protection against 1-methyl-4-phenyl-1,2,3,6-tetrahydropyridine (MPTP)-induced degeneration of the nigrostriatal dopaminergic pathway [26]; (3) antagonism of antipsychotic drug-induced vacuous chewing movements in the rat model of human tardive dyskinesia [27]; and (4) prevention of NMDA receptor antagonist (MK-801)-induced deficits in social interaction in rats [28].

In the initial design of the γ-lactam PLG peptidomimetic **2**, the isobutyl side chain of the leucyl residue was not incorporated into the structure, in order to simplify the synthesis. The potent activity of γ-lactam peptidomimetic **2** indicated that the isobutyl side chain was not an absolute requirement for modulating dopamine receptors. The synthesis of analogues of **2** in which lipophilic moieties were incorporated into the structure to mimic the isobutyl side chain of the leucyl residue of PLG yielded analogues **10**–**12** (Figure 2) with increased activity, suggesting that the lipophilic side chain was enhancing the binding of the compounds to the PLG binding site presumably by accessing a hydrophobic binding pocket [29–30]. A similar effect was seen with the substituted β-lactam analogue of PLG, compound **13**, developed by Palomo et al. [31].

Other scaffolds have been employed successfully to generate PLG peptidomimetics. A β-amino acid approach to the Freidinger lactams that employs the piperidin-2-one scaffold yielded the active PLG peptidomimetic **14** [32]. Another example is the pyridine-based PLG peptidomimetics developed by Saitton et al. [33–34] and illustrated by **15**. Peptidomimetic **15** cannot adopt a type II β-turn, but rather exists in an extended conformation. Thus, the activity seen with **15** is not consistent with the postulated type II β-turn bioactive conformation hypothesis that is supported by the lactam and other highly conformationally constrained PLG peptidomimetics that have been developed. It was suggested that **15** is interacting with the PLG modulatory binding site in a different manner than **2** and its analogues [33]. Possible support for such a hypothesis can be seen in the different activity profiles of α,α-disubstituted glycine analogues of PLG and the corresponding α,α-disubstituted derivatives of lactam PLG peptidomimetic **2** [35]. In addition, subsequent studies, as detailed below, have shown that conformationally restricted molecules constrained in different conformations, but capable of projecting the key pharmacophores in the same relative area of space, produce active PLG peptidomimetics.

### Design of photoaffinity labels to identify the PLG modulatory site

**Figure 3:**
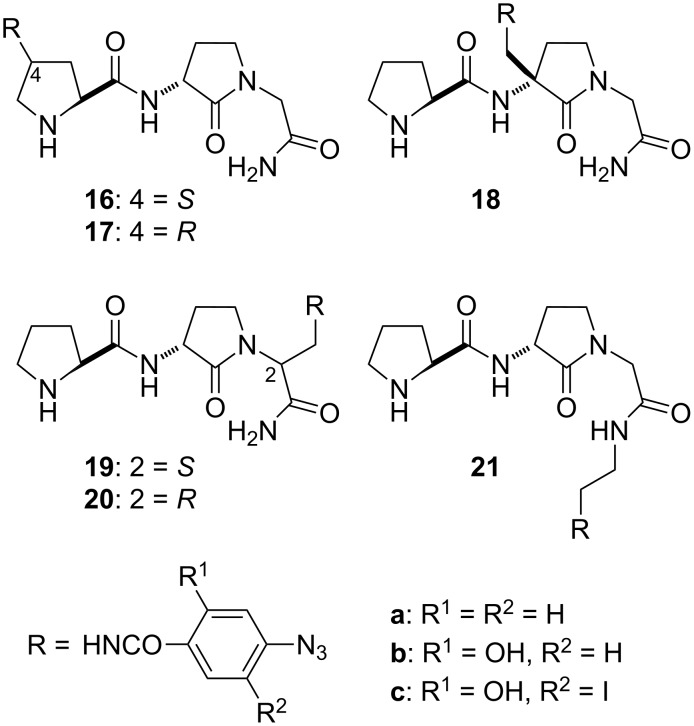
Lactam-based photoaffinity ligands of the PLG modulatory site.

γ-Lactam peptidomimetic **2** served as a useful platform on which to build ligands that proved useful in delineating the target at which PLG and its peptidomimetics act. A series of potential photoaffinity ligands was developed by placing a photoreactive moiety at different points about **2** (**16**–**21**, Figure 3) [3,36]. These photoaffinity ligands were found to retain the ability to modulate dopamine receptors to varying degrees, thus indicating that the incorporation of the photoreactive moieties did not have a significantly adverse effect on ligand binding to the modulatory site. Cross-linking of photoaffinity ligands **21b** and **21c** with the modulatory site gave a receptor preparation in which the dopamine receptor was modulated by the covalently linked photoaffinity ligand [3]. A radio-labeled form of photoaffinity ligand **16c** was used to demonstrate that the site at which PLG and its peptidomimetics act to produce their dopamine receptor modulatory effects is located on the dopamine receptor [9]. This represented the first direct evidence that PLG and its peptidomimetics were acting as allosteric modulators of the dopamine receptor.

### Spiro and bicyclic PLG peptidomimetics

The incorporation of a bridging unit into lactam **2** from C-3 to the adjacent amide bond nitrogen yields the spiro-based PLG peptidomimetics of type **B** (Figure 1). The spiro lactam scaffolds restrict the Φ_2_ and ψ_2_ torsional angles of a β-turn and depending upon the chirality of the central carbon atom these constraints can either mimic a type II or type II’ β-turn [37–40].

The insertion of a thiomethylene bridging unit from lactam C-5 to the acetamide α-carbon provides the bicyclic PLG peptidomimetics of type **C** (Figure 1). The result is a thiazolidine ring fused with the lactam ring. Although other types of bridging units have been employed in developing bicycle-based peptidomimetics, the thiomethylene unit is quite attractive as the synthesis into such systems is simplified, because the amino acid cysteine can be used. This bicyclic constraint was initially developed by Nagai and Sato [41]. In this constrained system, it is the ψ_2_ and Φ_3_ torsion angles that are constrained to values near those of an ideal type II β-turn as determined through computational calculations [42].

**Figure 4:**
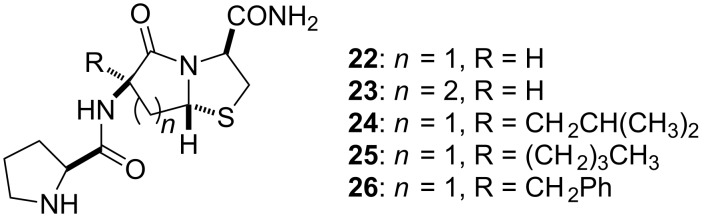
Bicyclic PLG peptidomimetics.

Of these two constrained systems, the bicyclic PLG peptidomimetics **22** and **23** (Figure 4) provided derivatives with significant dopamine-receptor-modulating activity [42]. As in the lactam series, the biological activity of the bicylic derivatives was enhanced by incorporating hydrophobic moieties of the bicyclic ring system that would be in a position to access the hydrophobic pocket believed to be interacting with the leucyl side chain of PLG, peptidomimetics **24**–**26** (Figure 4) [43].

### Spiro-bicyclic PLG peptidomimetics

**Figure 5:**
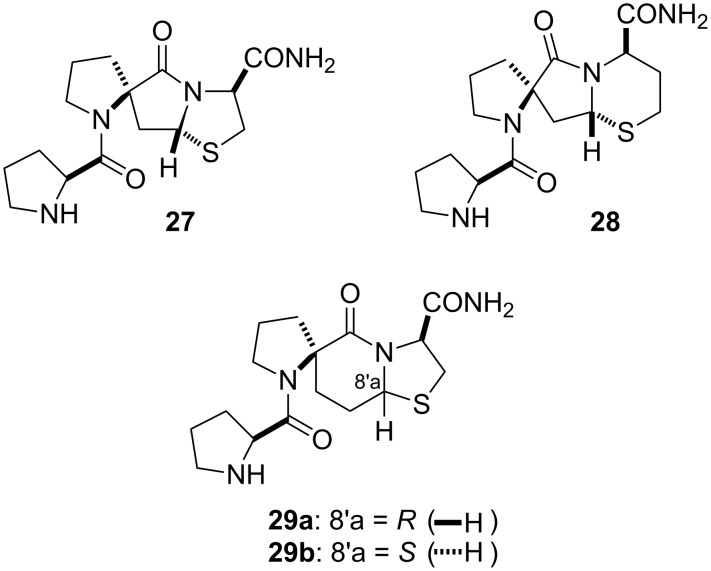
Spiro-bicyclic PLG peptidomimetics.

By combining the spiro and bicyclic constraints into a single structure, spiro-bicyclic PLG peptidomimetics of type **D** (Figure 1) were obtained. These highly restricted scaffolds constrain three of the four torsion angles that define a turn structure, Φ_2_, ψ_2_, and Φ_3_, making them among the best scaffolds at locking a peptide into a turn structure. The 5.5.5 spiro-bicyclic scaffold of peptidomimetic **27** (Figure 5) was found to mimic a type II β-turn as demonstrated through computational studies [44]. Peptidomimetic **27** was shown to enhance the binding of dopamine receptor agonists to the dopamine receptor in a manner similar to that of PLG [45]. Subsequently, it was shown that the 5.5.6 (**28**) and 5.6.5 (**29**) spiro-bicyclic scaffolds also served as excellent mimics of a type II β-turn [46]. In fact, peptidomimetics **28** and **29a** were more active than **27** in shifting the ratio of low and high affinity states of the dopamine receptor to the high affinity state and in enhancing apomorphine-induced rotations in the 6-hydroxydopamine-lesion rat model of Parkinson’s disease [46]. The results showed that modifying the ring sizes of the spiro-bicyclic scaffolds had a significant effect on the activity of the spiro-bicyclic peptidomimetic and because of the highly restricted nature of the spiro-bicyclic systems the results provided strong evidence in support of the hypothesis that the bioactive conformation of PLG was that of a type II β-turn.

**Scheme 1:**
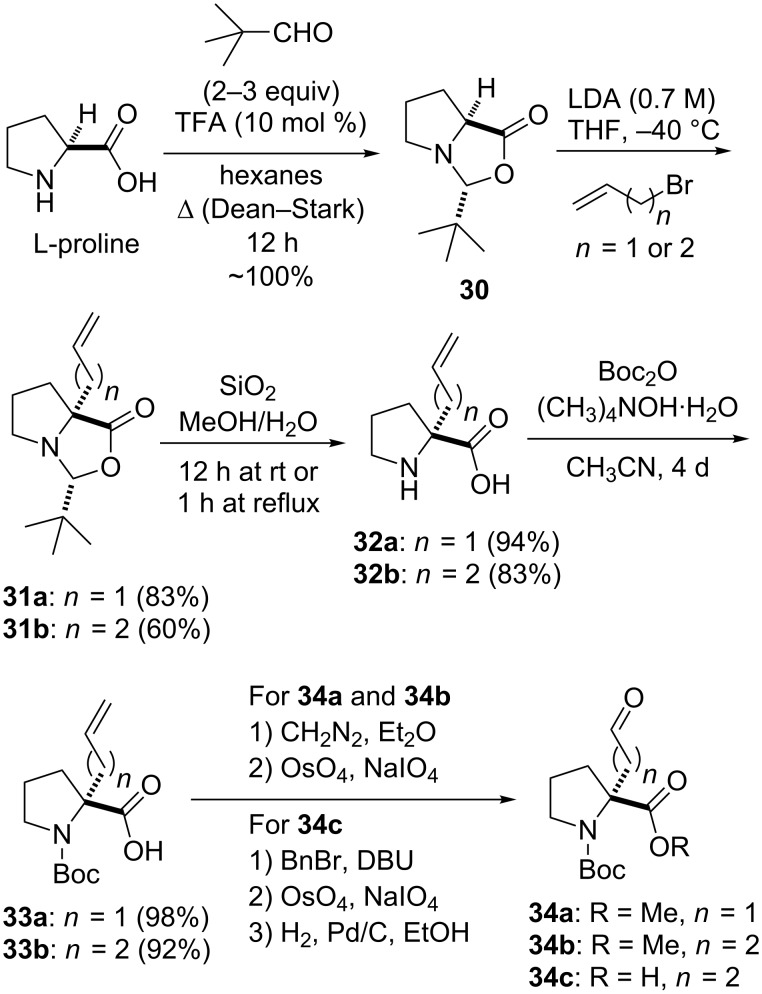
Synthesis of α-alkylaldehyde proline derivatives by Seebach's “self-regeneration of chirality” methodology.

The synthetic approach to the novel highly constrained spiro-bicyclic turn mimics found in PLG peptidomimetics **27**–**29** relied on α-alkylaldehyde proline derivatives (**34**, Scheme 1) as key starting materials [44,46]. These aldehyde intermediates were obtained from L-proline via the highly moisture-labile oxazolidinone **30** of Seebach's “self-regeneration of chirality” methodology [47]. Stereoselective alkylation of **30** provided alkylated oxazolidinone **31** and cleavage of this *N*,*O*-acetal provided α-alkylated proline **32**, which when Boc-protected gave **33**. The formation of **34a** and **34b** was accomplished by initial esterification of **33a** and **33b**, respectively, followed by oxidative cleavage of the double bond in each case with OsO_4_/NaIO_4_ [46]. An efficient and reproducible conversion of **33b** into **34c** was developed that consisted of the following three-step route: (1) benzyl ester formation, (2) oxidative cleavage of the double bond with OsO_4_/NaIO_4_, and (3) hydrogenolysis of the benzyl ester [36,48].

Although the Seebach methodology provides a highly stereoselective way to α-alkylated prolines, there are several shortcomings to the originally developed protocols [47]. In the oxazolidinone formation reaction, these shortcomings include the need for a large excess of the costly pivalaldehyde, long condensation reaction times of 3–4 days, and issues surrounding the isolation and handling of the moisture-sensitive oxazolidinone. By replacing pentane (capable of forming a 2% azeotrope with water) with hexanes (capable of forming a 6% azeotrope with water) it was found that the reaction time could be significantly reduced from several days to 12–18 hours and that the amount of pivalaldehyde could be reduced from six to two equivalents [49]. With respect to the *N*,*O*-acetal cleavage reaction, wherein **31** is converted to **32**, the hydrolysis of **31** was originally carried out under rigorous acidic conditions (aqueous HCl under reflux) and purification of the resulting α-alkylated proline **32** required tedious ion-exchange chromatography [47]. It was subsequently found that simply stirring a solution of the alkylated oxazolidinone **31** in a methanol/water solution with silica gel (200–400 mesh, 60 Å) either at room temperature for 12 hours [50] or under reflux for 1 hour [49], followed by simple filtration procedures, provided α-alkylated proline **32** in analytically pure form and in excellent yield. Protection of the amino group of **32** by using typical procedures gives lower yields than what is normally observed for this type of amine protection, probably due to steric factors resulting from the presence of the new fully substituted carbon center that has been introduced alpha to the amino group. However, Boc-protected α-alkylated proline derivatives could be obtained in yields greater than 90% through conversion of the α-alkylated proline to its salt form with tetramethylammonium hydroxide in CH_3_CN during the amine protection reaction [51].

**Scheme 2:**
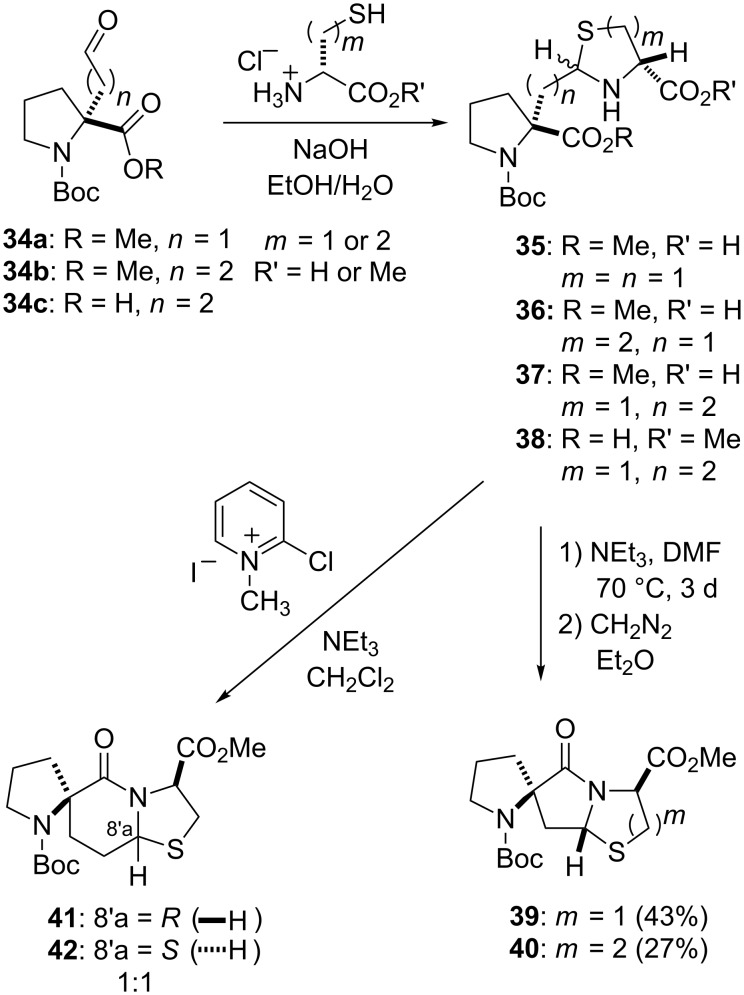
Synthetic approaches to the spiro-bicyclic scaffolds.

The construction of the spiro-bicyclic scaffolds was accomplished through the condensation of α-alkylaldehyde proline derivatives **34a**–**c** with either D-cysteine or D-homocysteine derivatives to give diastereoisomeric mixtures of thiazolidines **35**, **37** and **38** and thiazines **36**, respectively (Scheme 2). Formation of the lactam ring from the thiazine and thiazolidines to generate the spiro-bicyclic systems was accomplished by two different methods. In one approach, thermal cyclization of **35** and **36** followed by esterification gave the cyclized 5.5.5- and 5.5.6-spiro bicyclic products, **39** and **40**, respectively [44,46]. In this approach, only one of two possible diastereoisomeric products was obtained in each case. Thiazolidine **37** could not be cyclized under these conditions to generate the corresponding 5.6.5 spiro-bicyclic system. Instead, the 5.6.5 spiro-bicyclic system was obtained by activation of the free carboxylic acid of **38** with Mukaiyama’s reagent (2-chloro-1-methylpyridinium iodide (CMPI) [52]) followed by in situ lactam formation [46]. Under these advantageous kinetic conditions, two diastereoisomers of the 5.6.5 spiro-bicyclic system, **41** and **42**, were obtained in a 1:1 ratio. The two diastereoisomers vary only in the stereochemistry at C-8’a.

### PLG peptidomimetics mimicking type VI β-turn and polyproline II helix conformations

**Figure 6:**
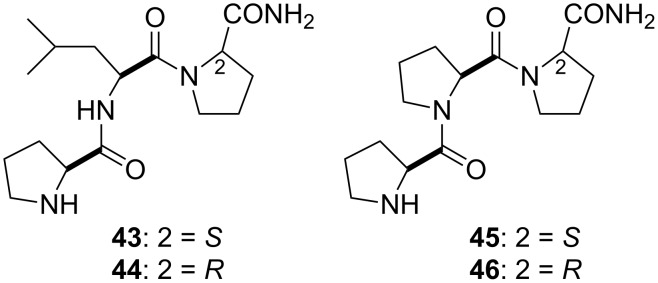
Prolyl PLG analogues.

Early SAR studies on PLG showed that when the glycinamide residue was substituted with both L-and D-prolinamide residues, active dopamine receptor modulating peptides **43** and **44** (Figure 6) were obtained [13]. Likewise, triproline analogues of PLG, peptides **45** and **46**, in which the C-terminal residue was either L-or D-prolinamide gave active compounds [53]. These results presented a paradox. It was expected that only those PLG analogues possessing a D-prolyl residue at the C-terminal end would show activity, as only those analogues would be capable of assuming the postulated type II β-turn bioactive conformation of PLG. Since the prolyl analogues possessing a C-terminal L-prolyl residue would not be able to assume a type II β-turn, the fact that they were active was not consistent with the hypothesis that the bioactive conformation of PLG is a type II β-turn. It was speculated that the prolyl derivatives with the C-terminal L-prolyl residue are capable of adopting a conformation that places key pharmacophore moieties in the same relative topological space that these moieties occupy in the peptidomimetics constrained to a type II β-turn [36,48–49].

**Figure 7:**
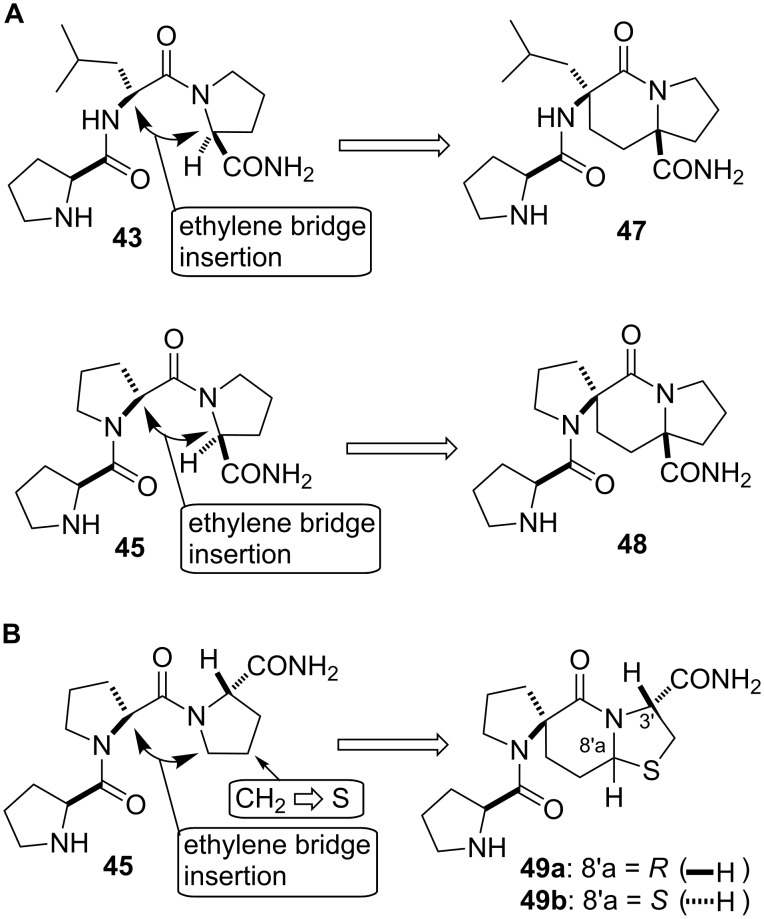
(**A**) Type VI β-turn mimics. An ethylene bridge connection in **43** and **45** between the α-carbon of the second residue and the α-carbon of the C-terminal prolyl residue that is in a *cis*-amide bond configuration yields spiro-bycyclic PLG peptidomimetics **47** and **48** constrained in a type VI β-turn conformation. (**B**) Polyproline II helix mimics. An ethylene bridge connection in **45** between the α-carbon of the second prolyl residue and the δ-carbon of the C-terminal prolyl residue that is in a *trans*-amide bond configuration, and the replacement of the γ-CH_2_ with S yields spiro-bicyclic PLG peptidomimetics **49a** and **49b** in a polyproline II helix conformation.

Analysis of possible conformations that the homochiral prolyl peptides could assume indicated two possibilities, as depicted in Figure 7. In one case, the presence of a prolyl residue introduces the possibility of a *cis*-amide bond at the C-terminus with the result being a type VI β-turn conformation. To test this hypothesis, Germanas’ indolizidinone scaffold [54] was employed as the type VI β-turn conformation mimic to design **47** and **48** (Figure 7A) [49,55]. A second possibility was that triproline **45** could assume a polyproline II helix conformation, wherein all the Pro–Pro amide bonds are in a *trans*-configuration. For testing this hypothesis, the spiro-bicyclic peptidomimetics **49a** and **49b** were designed to mimic the polyproline II helix conformation (Figure 7B) [48,56]. These two spiro-bicyclic peptidomimetics are C-3’ epimers of the spiro-bicyclic type II β-turn mimics **29a** and **29b**.

**Scheme 3:**
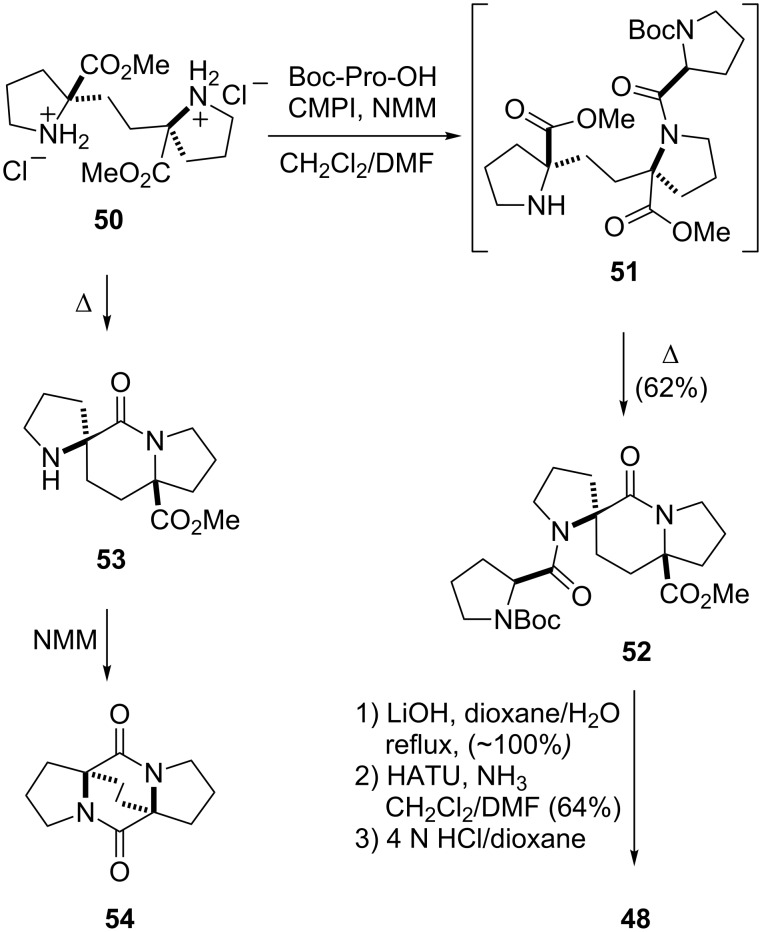
Synthesis of spiro-bicyclic type VI β-turn mimic **48**.

In the retrosynthetic analysis of spiro-bicyclic type VI β-turn mimic **48**, a disconnection at the lactam bond in the indolizidinone core was envisioned, which would give rise to a symmetric unit of two prolines linked enantioselectively via a two-carbon linkage at their α-carbons [55]. Such an approach proved successful, as shown in Scheme 3. The ethylene-linked biproline derivative **50** was readily converted to spiro-bicyclic **52** under conditions wherein the CMPI-activated ester of Boc-Pro-OH was coupled to **50** to give the acylated biproline intermediate **51**, which when heated cyclized to **52** [55]. The sterically congested nature of the spiro indolizidinone scaffold, due in part to its boat-shaped conformation, was illustrated by the observation that when **50** was converted to spiro indolizidinone **53**, this material could not be efficiently acylated with Boc-Pro-OH under a variety of coupling conditions. Instead, **53** had a propensity to convert to the diketopiperazine **54**. Also, the methyl ester of **52** resisted direct amidation under a variety of standard conditions. Rather, **52** had to be hydrolyzed to the acid and then coupled to NH_3_ with 2-(1*H*-7-azabenzotriazol-1-yl)-1,1,3,3-tetramethyluronium hexafluorophosphate (HATU) as the coupling reagent in order to provide the primary carboxamide intermediate, which could be deprotected to give **48**.

**Scheme 4:**
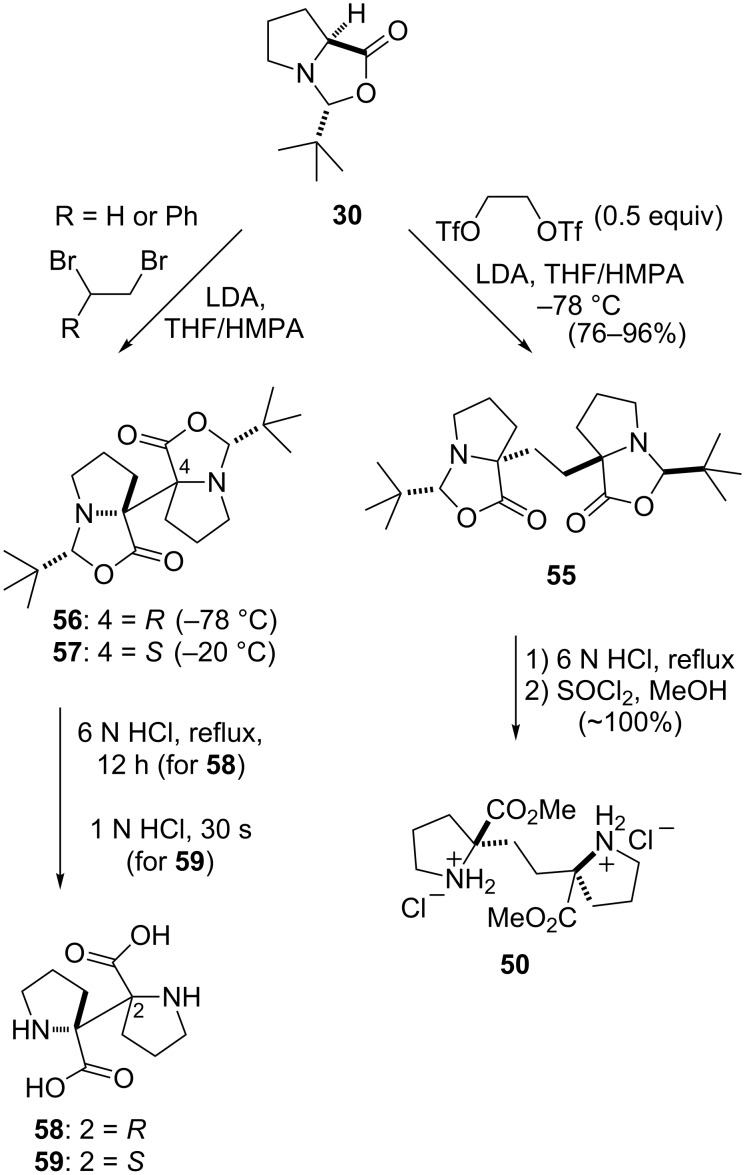
Biproline formation from Seebach’s oxazolidinone.

The lithium enolate of Seebach’s oxazolidinone **30** served as the starting material for the stereoselective synthesis of ethylene-linked biproline derivative **50**. The nature of the bis-electrophile proved to be crucial to the outcome of the alkylation reaction (Scheme 4). The alkylation of **30** with either symmetric (1,2-dibromoethane) or asymmetric (1,2-dibromo-1-phenylethane) vicinal dihalides resulted in the efficient dimerization of **30** to afford the biprolyl oxazolidinones **56** and **57**, which served as precursors to the corresponding novel (*R*,*R*)-α,α’-biproline **58** and *meso*-α,α’-biproline **59** [57]. In contrast, the reaction of the more electrophilic alkylating agent glycol bistriflate with the enolate of **30** provided the desired dimer **55**, which after acid hydrolysis of the dimer followed by Fischer esterification yielded **50** [55].

The type VI β-turn PLG peptidomimetics **47** and **48** and the spiro-bicyclic polyproline II helix PLG peptidomimetic **49a** were shown to possess positive dopamine-receptor modulatory activity as demonstrated by their ability to enhance the binding of the dopamine receptor agonist *N*-propylnorapomorphine (NPA) to dopamine D_2_ receptors [48–49]. These results provide evidence for the hypothesis that the homochiral prolyl peptides **43** and **45** are able to adopt either a type VI β-turn or a polyproline II helix as their bioactive conformation. It is postulated that these highly constrained molecules are able to produce a modulatory response because each can present the necessary topological arrangement of key pharmacophore moieties in a manner similar to that of PLG peptidomimetics that are restricted to a type II β-turn conformation. A comparison of the Φ, ψ, and ω torsion angles of the type II β-turn, the type VI β-turn, and the polyproline II helix conformational types indicates that they each possess similar torsion angles at their N-termini, but they differ at their C-termini. However, they all place the carboxamide NH_2_ pharmacophore in the same relative position in space [48–49].

### Peptidomimetic negative modulators of the D_2_ dopamine receptor

Biological evaluation of the C-8’a epimer of the type II β-turn PLG peptidomimetic **29a**, spiro-bicyclic **29b**, and of the C-8’a epimer of the polyproline II helix PLG peptidomimetic **49a**, spiro-bicyclic **49b**, revealed that the **29b** and **49b** diastereoisomers were not positive modulators of the D_2_ dopamine receptors, but rather were negative modulators of the receptor as they decreased the binding of the dopamine receptor agonist *N*-propylnorapomorphine to the receptor [48]. The fact that all four peptidomimetics were capable of displacing a PLG peptidomimetic radioligand in a competitive binding assay indicated that the positive and negative modulators are interacting with the same allosteric site on the D_2_ dopamine receptor. The structural difference between **29a** and **29b** and between **49a** and **49b** is the chirality at the C-8’a bridgehead carbon atom. This difference, it was postulated, has an effect on the conformation adopted by the thiazolidine ring within the spiro-bicyclic scaffold. In particular, modelling studies suggested that the pucker of the thiazolidine ring in negative modulators **29b** and **49b** caused the C-2’ carbon to occupy a different area of topological space than this carbon occupies in the positive modulators **29a** and **49a** [48].

**Figure 8:**
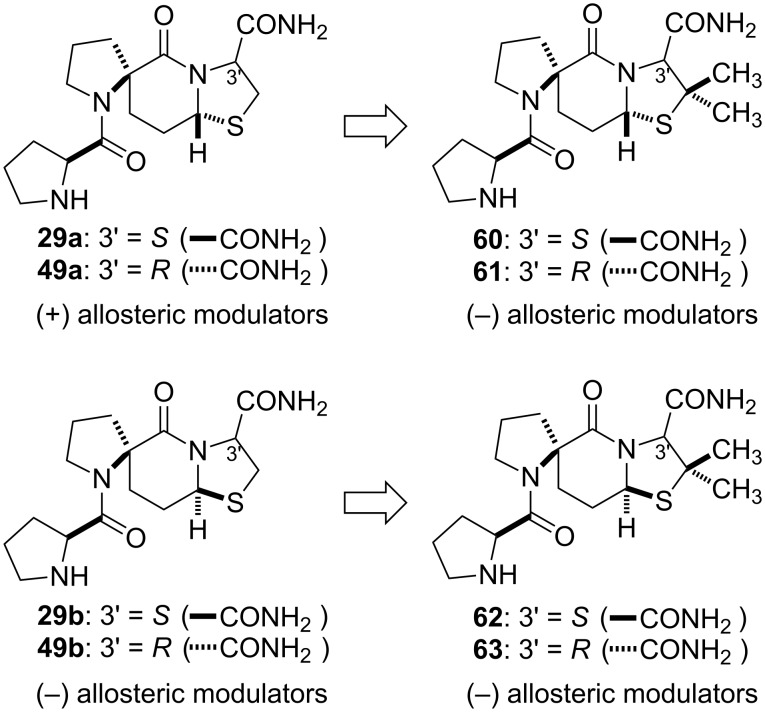
Positive and negative allosteric modulators of the D_2_ dopamine receptor based on the 5.6.5 spiro-bicyclic scaffold.

It was speculated that the different conformational effects between the negative and positive allosteric modulators translated into different conformational changes when these ligands bound to the allosteric binding site. This in turn produced different conformational effects at the orthosteric site where the dopamine receptor agonists bind. To test this hypothesis, the syntheses of β-dimethyl derivatives of **29a**, **29b**, **49a**, and **49b**, i.e., spiro-bicyclic peptidomimetics **60**–**63** (Figure 8), respectively, were carried out [58].

In the case of the positive allosteric modulators, **29a** and **49a**, we postulated that by placing the dimethyl groups on the C-2’ carbon of the thiazolidine ring to give the corresponding analogues **60** and **61**, we would be placing steric bulk in the same region of topological space occupied by the C-2’ carbon in the different pucker conformation of the negative modulators **29b** and **49b**. It was predicted that the dimethyl substitution on a positive allosteric modulator would convert it into a negative allosteric modulator. The dimethyl analogues, **60** and **61** showed significant negative modulatory activity, as demonstrated by their ability to negatively affect the binding of the dopamine receptor agonist NPA to the D_2_ receptor and to shift the EC_50_ value for [^3^H]NPA binding to dopamine D_2_ receptors by 2.7- and 2.8-fold, respectively, compared to the control [58]. Thus, both compounds decreased the affinity of the agonist NPA to the D_2_ dopamine receptor. The results supported the proposition that the introduction of dimethyl groups into the structure of the positive modulators resulted in molecules that resembled the conformational characteristics of the unsubstituted negative allosteric modulators.

The introduction of dimethyl groups into the structure of the negative modulators **29b** and **49b** gave analogues **62** and **63**, respectively. These analogues also exhibited negative allosteric modulatory activity, albeit at a lower level than the unsubstituted peptidomimetics. Molecular models of **49b** and **63**, for example, show that the thiazolidine C-2’ carbons of these two molecules overlay quite well, but that the methyl groups of **63** now occupy topographical space outside that occupied by the thiazolidine C-2’ carbon of **49b**. It was speculated that this may produce adverse steric effects and that this may be responsible for the observed weaker activity seen with **62** and **63**.

## Conclusion

The development of PLG peptidomimetic probes has proved valuable in helping to elucidate the structural and molecular mechanism by which an endogenous neuropeptide, PLG, modulates dopaminergic neurotransmission. This knowledge will be useful in developing novel central nervous system (CNS) drugs to treat conditions in which the dopamine receptors are directly implicated (i.e., Parkinson’s disease, schizophrenia, Gilles de la Tourette syndrome, etc.) [59–61]. Within the context of G-protein coupled receptors, this work illustrates the potential value of receptor modulation as a means of perturbing traditional ligand–receptor interaction [62–63] and it demonstrates that this can be a successful platform for understanding biological function with peptidomimetic probes.
